# Induction of methionine adenosyltransferase 2A in tamoxifen-resistant breast cancer cells

**DOI:** 10.18632/oncotarget.5298

**Published:** 2015-09-18

**Authors:** Nguyen Thi Thuy Phuong, Sang Kyum Kim, Ji Hye Im, Jin Won Yang, Min Chang Choi, Sung Chul Lim, Kwang Yeol Lee, Young-Mi Kim, Jeong Hoon Yoon, Keon Wook Kang

**Affiliations:** ^1^ College of Pharmacy and Research Institute of Pharmaceutical Sciences, Seoul National University, Seoul 151-742, South Korea; ^2^ College of Pharmacy, Chungnam National University, Daejeon 305-764, South Korea; ^3^ Department of Pathology, College of Medicine, Chosun University, Gwangju 501-759, South Korea; ^4^ College of Pharmacy, Chonnam National University, Gwangju 500-757, South Korea; ^5^ College of Pharmacy, Hanyang University, Ansan 426-791, South Korea; ^6^ Department of Oral & Maxillofacial Pathology, College of Dentistry, Daejeon Dental Hospital, Wonkwang University, Daejeon 302-120, South Korea

**Keywords:** tamoxifen resistance, miR-146b, MAT2A, NF-κB, PTEN

## Abstract

We previously showed that *S*-adenosylmethionine-mediated hypermethylation of the *PTEN* promoter was important for the growth of tamoxifen-resistant MCF-7 (TAMR-MCF-7) cancer cells. Here, we found that the basal expression level of methionine adenosyltransferase 2A (MAT2A), a critical enzyme for the biosynthesis of *S*-adenosylmethionine, was up-regulated in TAMR-MCF-7 cells compared with control MCF-7 cells. Moreover, the basal expression level of MAT2A in T47D cells, a TAM-resistant estrogen receptor-positive cell line was higher compared to MCF-7 cells. Immunohistochemistry confirmed that MAT2A expression in TAM-resistant human breast cancer tissues was higher than that in TAM-responsive cases. The promoter region of human *MAT2A* contains binding sites for nuclear factor-κB, activator protein-1 (AP-1), and NF-E2-related factor 2 (Nrf2), and the activities of these three transcription factors were enhanced in TAMR-MCF-7 cells. Both the protein expression and transcriptional activity of MAT2A in TAMR-MCF-7 cells were potently suppressed by NF-κB inhibition but not by c-Jun/AP-1 or Nrf2 knock-down. Interestingly, the expression levels of microRNA (miR)-146a and -146b were diminished in TAMR-MCF-7 cells, and miR-146b transduction decreased NF-κB-mediated MAT2A expression. miR-146b restored PTEN expression via the suppression of *PTEN* promoter methylation in TAMR-MCF-7 cells. Additionally, miR-146b overexpression inhibited cell proliferation and reversed chemoresistance to 4-hydroxytamoxifen in TAMR-MCF-7 cells.

## INTRODUCTION

Breast cancer is one of the most common malignancies in Western countries. Because estrogen is essential for either mammary gland or breast cancer development, the administration of anti-estrogens to suppress breast tumor growth is an important therapeutic option against estrogen receptor-positive breast cancer [1]. Tamoxifen (TAM), a non-steroidal anti-estrogen, has been most widely used for chemotherapy or chemopreventive purposes in breast cancer patients. However, TAM resistance acquisition is a common problem in anti-estrogen therapy [2], and the resistance mechanism remains unsolved.

Methionine adenosyltransferase (MAT), encoded by the genes *MAT1A* and *MAT2A* [3], is an essential enzyme for the survival of an organism; it is the only enzyme responsible for the formation of *S*-adenosylmethionine (SAM), a principal biological methyl donor [3–5]. The physiological roles of MAT1A and MAT2A have been mainly studied in the liver. MAT1A, which is mostly expressed in the liver, is a marker of a normal differentiated or mature liver phenotype [4]. However, MAT2A is frequently found in fetal liver and is gradually replaced by MAT1A during liver development. In contrast to MAT1A, MAT2A is believed to be a marker for rapid growth and dedifferentiation [4]. In hepatocytes, increased MAT2A expression is associated with malignant degeneration and uncontrolled tumor growth [4, 6–9]. Additionally, SAM produced by MAT is a key metabolite regulating hepatocyte growth and differentiation [8]. Outside of the liver, MAT2A and SAM expression has been reported in activated T lymphocytes [10] and in human colon cancer cells where MAT2A expression is required for mitogen-induced cell growth [11]. However, regulation of MAT2A expression in TAM-resistant breast cancer and its roles in tumor growth and chemoresistance have been uncovered. In our previous study, the cellular levels of SAM and expression of DNA methyltransferase 1 (DNMT1, a key enzyme in DNA methylation) were shown to be up-regulated in TAM-resistant MCF-7 (TAMR-MCF-7) cells [12]. Those cellular events cause hypermethylation of the *PTEN* promoter, leading to decreased PTEN expression and sustained activation of phosphoinositide 3-kinase (PI3K) [12]. Hence, we first examined the expression levels of MAT2 in TAMR-MCF-7 cells. As expected, we found that MAT2 expression was up-regulated in TAMR-MCF-7 cells compared with control MCF-7 cells. Moreover, MAT2 expression was more frequent in TAM-resistant human breast cancer tissues than in TAM-responsive cases.

In liver cancer, the up-regulation of MAT2A occurs via transcriptional activation [13]. The promoter region of human *MAT2A* contains several functional binding sites for transcription factors, including nuclear factor-κB (NF–κB), activator protein-1 (AP–1), NF-E2 related factor 2 (Nrf 2), and specific protein1 (Sp1) [13]. In the present study, we attempted to elucidate the transcriptional control of MAT2A in TAMR-MCF-7 cells and found that NF-κB activation via microRNA (miR)-146b down-regulation stimulated MAT2A gene transcription. We also found that miR-146b overexpression recovered PTEN down-regulation and 4-hydroxytamoxifen responsiveness, and significantly inhibited the proliferation of TAMR-MCF-7 cells.

## RESULTS

### Up-regulation of MAT2A expression in TAMR-MCF-7 cells

**Figure 1:**
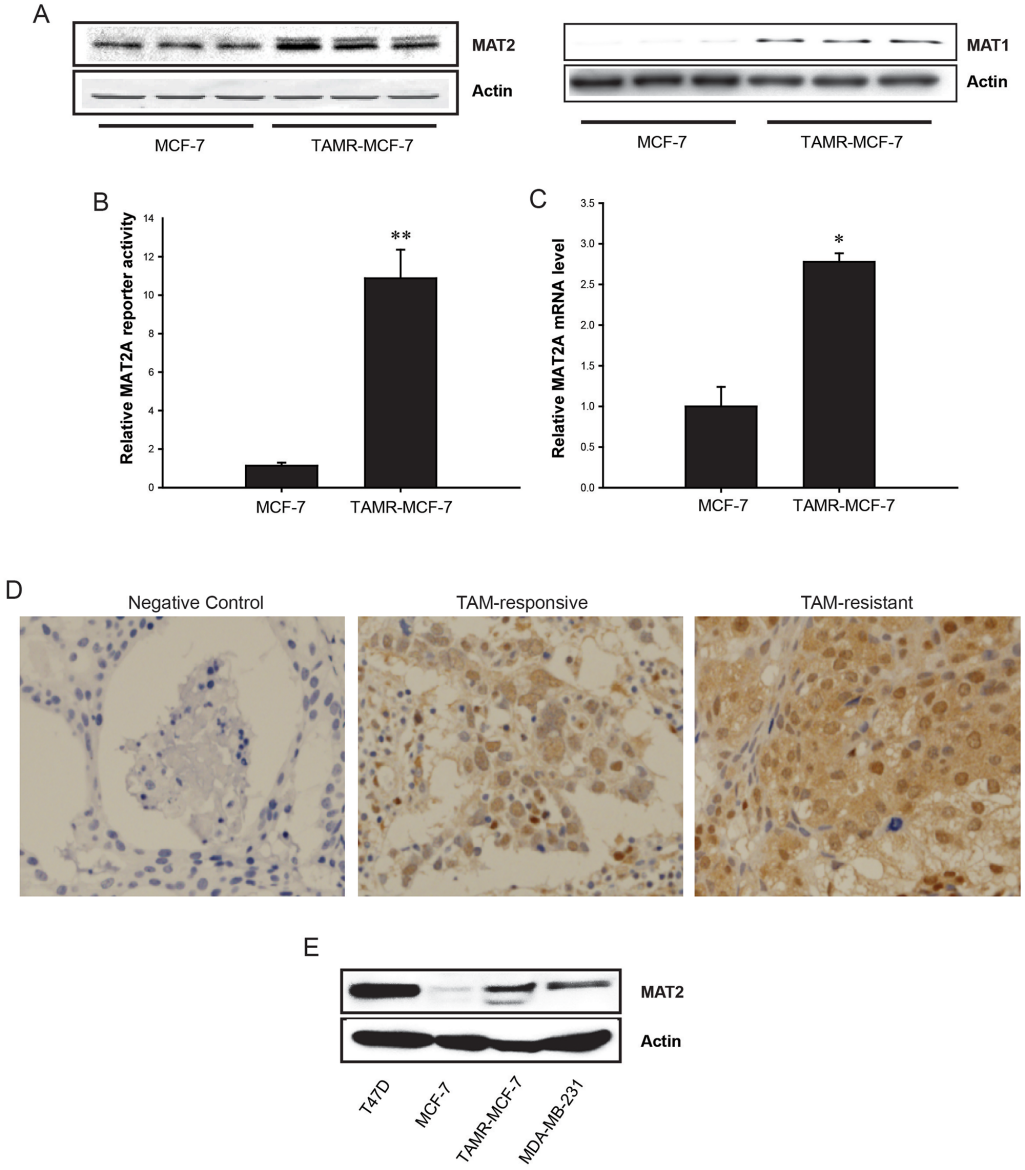
MAT2A expression in MCF-7 and TAMR-MCF-7 cells **A.** Immunoblot analysis of MAT2 in MCF-7 and TAMR-MCF-7 cells. Each lane represents different sample. **B.** Basal MAT2A promoter reporter activities in MCF-7 and TAMR-MCF-7 cells. MCF-7 and TAMR-MCF-7 cells were seeded in 12 wells plate for 1 day. Both the cell types (60% confluency) were then transiently co-transfected with MAT2A-luc reporter plasmid containing −570/+61 bp human MAT2A promoter (1 μg/well) and phRL-SV (hRenilla) (1 ng/well). Dual luciferase reporter assays were performed on the lysed cells 18 h after transfection in serum free condition. Reporter gene activity was calculated as a relative ratio of firefly luciferase to hRenilla luciferase activity. Data represent mean ± SD with 6 different samples (significant versus MCF-7 cells, **P < 0.01). **C.** MAT2A mRNA levels in MCF-7 and TAMR-MCF-7 cells. MAT2A mRNA levels were determined by quantitative RT-PCR. Data represent the mean ± SD (*n* = 4)(significant versus MCF-7 cells, **P < 0.01). **D.** Immunohistochemistry of MAT2A in human breast cancer tissues. Four TAM-responsive and four TAM-resistant cases were estimated. The brown color staining represents MAT2A expression. When we determined immunoreactivity in IgG-incubated breast cancer tissue samples (negative control), we could not detect any positive staining. **E.** MAT2A immunoblot analyses in T47D cells. The basal MAT2A levels were compared in T47D, MCF-7, TAMR-MCF-7 and MDA-MB-231 cells.

We previously revealed that the level of SAM was significantly enhanced in TAMR-MCF-7 cells compared with MCF-7 cells [12]. Because MAT enzymes, including MAT1A and MAT2A are involved in SAM synthesis, we determined the MAT1 and MAT2A expression levels in control MCF-7 and TAMR-MCF-7 cells using Western blot analysis. MAT2A protein levels were distinctly higher in TAM-MCF-7 cells than in MCF-7 cells (Figure 1A, left); Although the basal protein level of MAT1 was extremely low in MCF-7 cells, the protein expression was also enhanced in TAMR-MCF-7 cells (Figure 1A, right). Reporter gene analysis using a MAT2A-luc reporter plasmid containing a luciferase reporter and *a* −570/+61-bp human MAT2A promoter showed that MAT2A-luc reporter activity was enhanced in TAMR-MCF-7 cells (Figure 1B), suggesting that the enhanced MAT2A expression resulted from transcriptional activation of MAT2A. Quantitative real-time PCR also confirmed that MAT2A mRNA was abundant in TAMR-MCF-7 cells (Figure 1C). Moreover, we assessed the expression level of MAT2A in human breast cancer tissues by immunohistochemistry. Tumor tissues were obtained from two groups of patients, Four cases included in “Non-recurrence group after TAM therapy (TAM-responsive group)” experienced no recurrence for at least 6 years of follow-up after mastectomy with adjuvant TAM therapy. The other four cases in “Recurrence group after TAM therapy (TAM-resistant group)” relapsed within 3 to 4 years after mastectomy with adjuvant TAM therapy. Intensity of cytoplasmic MAT2A staining was scored by a certified pathologist, and the score is 1.25 ± 0.50 (TAM-responsive) and 2.50 ± 0.58 (TAM-resistant, *P* < 0.05), respectively (Figure 1D). We also analyzed Gene Expression Omnibus (GEO, http://www.ncbi.nlm.nih.gov/geo) data. The accession number was GSE32988, providing 62 pre-chemotherapy biopsies of HER2 normal breast cancer patients (ER-positive and ER-negative subtypes) with the results of the TAM-chemotherapy. Series matrix file was matched the probes on platform GLP96, excluded 5 normal samples, rearranged into groups according to existence of residual invasive cancer and normalized by a control gene. Interestingly, residual invasive cancer cases (TAM-resistant) showed the increasing tendency of MAT2A expression compared to no invasive cancer cases (*P* = 0.066) (Supplemental 1).

Although MCF-7 and T47D cells are the representative ER-positive breast cancer cell lines, T47D cells are relatively more resistant to TAM than MCF-7 clone [20]. Moreover, long-term incubation with 17-β-estradiol causes 60% increase in MCF-7 cell numbers over controls, while having no effect on growth of the T47D cell line [21]. When we assessed protein levels of MAT2A, the basal expression level of MAT2A in T47D cells was higher compared to MCF-7 and ER-negative MDA-MB-231 cells (Figure 1E).

### Roles of AP-1, NF-κB, and Nrf 2 in the up-regulation of MAT2A in TAMR-MCF-7 cells

**Figure 2:**
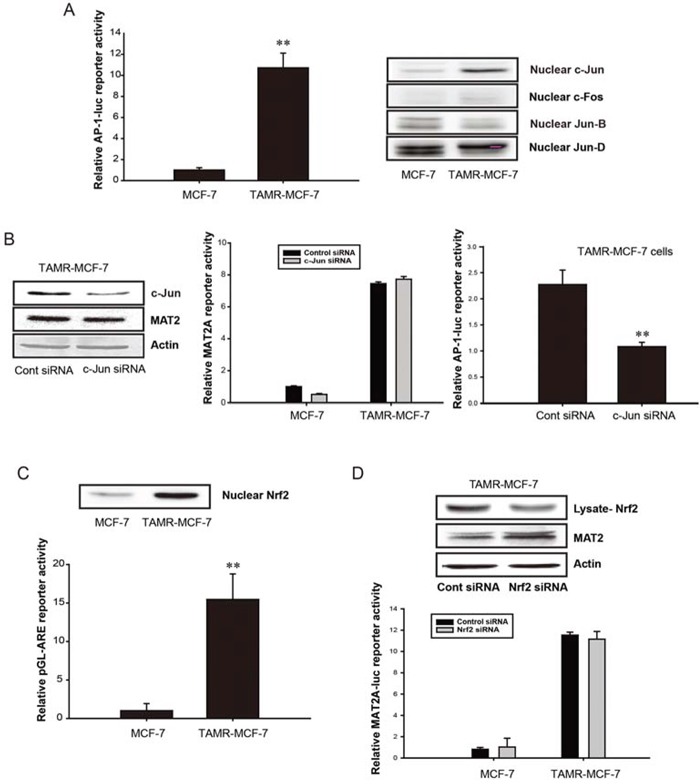
No involvement of AP-1 and Nrf2 in up-regulation of *MAT2A* gene in TAMR-MCF-7 cells **A.** AP-1 activities in MCF-7 and TAMR-MCF-7 cells. Left; AP-1 minimal reporter activity. MCF-7 and TAMR-MCF-7 cells were co-transfected with pAP-1-Luc reporter (1 μg/well) and phRL-SV plasmids (hRenilla, 1 ng/well). Luciferase reporter activity was determined described as figure legend of Figure 1B. Data represent mean ± SD with 6 different samples (significant versus MCF-7 cells, **P < 0.01). Right; Nuclear levels of c-Jun, c-Fos, Jun-B and Jun-D in MCF-7 and TAMR-MCF-7 cells. Each AP-1 protein was detected in the nuclear fractions isolated from serum-deprived MCF-7 and TAMR-MCF-7 cells. **B.** Left; Effect of c-Jun siRNA on MAT2A expression level in TAMR-MCF-7 cells. TAMR-MCF-7 cells were transfected with control or c-Jun siRNA (60 *p*mole/well) for 36 h. The protein levels of MAT2 and c-Jun in total cell lysates were determined by immunoblotting. Middle; Effect of c-Jun siRNA on *MAT2A* gene transcription in MCF-7 and TAMR-MCF-7 cells. Both cell lines were co-transfected with MAT2A-luc reporter plasmid containing −570/+61 bp human MAT2A promoter (1 μg/well), phRL-SV (1 ng/well) and control or c-Jun siRNA (20 *p*mole/well) for 36 h. Luciferase reporter activity was determined described as figure legend of figure 1B. Data represent mean ± SD with 3 different samples. Right; Effect of c-Jun siRNA on AP-1 reporter activity in TAMR-MCF-7 cells. TAMR-MCF-7 cells were co-transfected with control or c-Jun siRNA (20 *p*mole/well) and pAP-1-Luc (1 μg/well)/phRL-SV (1 ng/well) for 36 h. Data represent mean ± SD with 6 different samples (significant versus control siRNA-transfected TAMR-MCF-7 cells, **P < 0.01). **C.** Nrf2/ARE activities in MCF-7 and TAMR-MCF-7 cells. Upper; nuclear level of Nrf2 were detected in the nuclear fractions isolated from serum-deprived MCF-7 and TAMR-MCF-7 cells. Lower; the basal ARE reporter activities in MCF-7 and TAMR-MCF-7 cells. Both cell lines were transiently co-transfected with pGL-ARE-luc plasmid (1 μg/well) and phRL-SV (1 ng/well). Dual luciferase reporter assays were performed on the lysed cells 18 h after transfection in serum free condition. Data represent mean ± SD with 6 different samples (significant versus MCF-7 cells, **P < 0.01). **D.** Effect of Nrf2 siRNA on MAT2A expression (upper) and *MAT2A* gene transcription (lower) in TAMR-MCF-7 cells. Upper; MCF-7 cell and TAMR-MCF-7 cells were transfected with control or Nrf2 siRNA (60 *p*mole/well) for 36 h. MAT2 and Nrf2 protein expression was determined by immunoblotting. Lower; effect of Nrf2 siRNA on *MAT2A* gene transcription in MCF-7 and TAMR-MCF-7 cells. Both cell lines were co-transfected with MAT2A-luc reporter plasmid containing −570/+61 bp human MAT2A promoter (1 μg/well), phRL-SV (1 ng/well) and control or Nrf2 siRNA (20 *p*mole/well) for 36 h. Data represent mean ± SD with 3 different samples.

NF-κB and AP-1 are required for basal MAT2A expression and the tumor necrosis factor-α (TNF-α)-induced transactivation of MAT2A in human hepatoma cells [22]. Additionally, the Nrf2-binding antioxidant response element (ARE) region is located near the AP-1 and NF-κB binding sites in the promoter region of human MAT2A [22]. Here, we found that the minimal AP-1 reporter activity in TAMR-MCF-7 cells was greater than that in MCF-7 cells (Figure 2A, left). We then assessed the nuclear levels of c-Jun, c-Fos, Jun-B, and Jun-D in both cell types to identify the major form responsible for the elevation in AP-1 activity. Nuclear c-Jun level was selectively increased in TAMR-MCF-7 cells (Figure 2A, right). When TAMR-MCF-7 cells were transfected with c-Jun small interfering RNA (siRNA), AP-1 reporter activity was diminished; however, MAT2A expression and MAT2A-luc reporter activity were unchanged (Figure 2B). Additionally, both the minimal ARE reporter activity and nuclear Nrf2 level were enhanced in TAMR-MCF-7 cells compared with those in control MCF-7 cells (Figure 2C). To evaluate the relationship between Nrf2 activation and MAT2A induction in TAMR-MCF-7 cells, Nrf2 siRNA was introduced. However, the knockdown of Nrf2 did not decrease the protein level of MAT2A (Figure 2D, upper) or the enhanced reporter activity of MAT2A-luc in TAMR-MCF-7 cells (Figure 2D, lower). These data indicate that consistent activation of c-Jun/AP-1 and Nrf2 is not related to the increase in *MAT2A* gene transcription in TAMR-MCF-7 cells.

**Figure 3:**
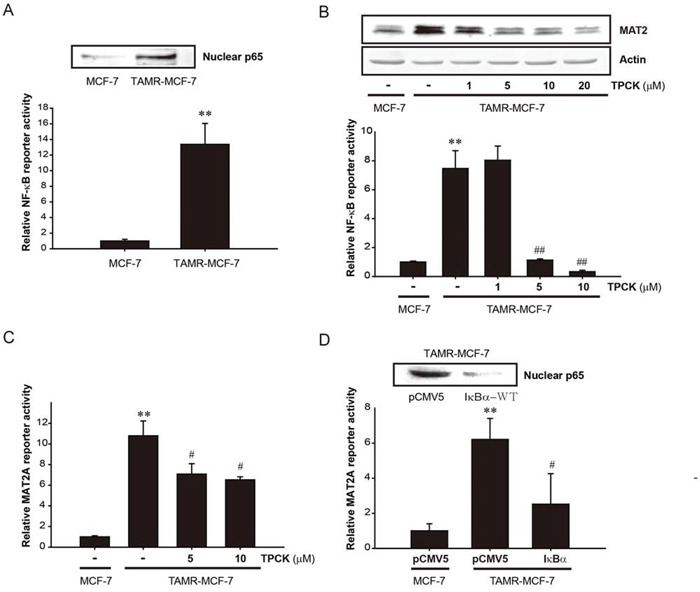
Role of NF-κB in up-regulation of *MAT2A* gene in TAMR-MCF-7 cells **A.** NF-κB activation in TAMR-MCF-7 cells. Upper; Nuclear level of p65. p65 protein levels were detected in the nuclear fractions isolated from serum-deprived MCF-7 and TAMR-MCF-7 cells. Lower; NF-κB minimal reporter activity. MCF-7 and TAMR-MCF-7 cells in 12 well plates were co-transfected with NF-κB-luc reporter (1 μg/well) and phRL-SV plasmids (1 ng/well) for 18 h in serum free condition. Luciferase reporter activity was determined described as figure legend of Figure 1B. Data represent mean ± SD with 6 different samples (significant versus MCF-7 cells, **P < 0.01). **B.** Effects of TPCK (NF-κB inhibitor) on MAT2A expression (upper) and NF-κB reporter activity (lower) in TAMR-MCF-7 cells. Upper; TAMR-MCF-7 cells were exposed to TPCK with the indicated concentration for 24 h. MAT2 expression level was determined from total cell lysates using immunoblotting. Lower; MCF-7 and TAMR-MCF-7 cells were co-transfected with NF-κB-luc reporter (1 μg/well) and phRL-SV plasmids (1 ng/well). Concomitantly, TPCK with the indicated concentration was treated to the transfected TAMR-MCF-7 cells for 24 h. Luciferase reporter activity was determined described as figure legend of figure 1B. Data represent mean ± SD with 3 different samples (significant versus MCF-7 cells, **P < 0.01; significant versus vehicle-treated TAMR-MCF-7 cells, ^##^*P* < 0.01). **C.** Effect of TPCK on *MAT2A* gene transcription. MCF-7 and TAMR-MCF-7 cells were co-transfected with MAT2A-luc reporter plasmid (1 μg/well) and phRL-SV plasmids (1 ng/well). Concomitantly, TPCK with the indicated concentration was treated to the transfected TAMR-MCF-7 cells for 24 h. Data represent mean ± SD with 3 different samples (significant versus MCF-7 cells, **P < 0.01; significant versus vehicle-treated TAMR-MCF-7 cells, ^#^*P* < 0.05). **D.** Effect of IκBα overexpression on the nuclear expression of p65 and *MAT2A* gene transcription in TAMR-MCF-7 cells. TAMR-MCF-7 cells were co-transfected with MAT2A-luc reporter plasmid (1 μg/well) and pCMV5 or IκBα overexpression plasmid (0.5 μg, respectively), and phRL-SV plasmids (1 ng/well) for 18 h in serum free condition. Data represent mean ± SD with 4 different samples (significant versus pCMV5-transfected MCF-7 cells, **P < 0.01; significant versus pCMV5-transfected TAMR-MCF-7 cells, ^#^*P* < 0.05).

The regulatory role of NF-κB in MAT2A expression was reported in several cancer types. p65 knockdown reduces the basal level of MAT2A expression and blocks the additive effect of IGF-1 on MAT2A in human colon cancer cells [23]. NF-κB activation is required for the induction of MAT2A by TNF-α in hepatoma cells [22]. In the present study, the nuclear p65 level and basal NF-κB reporter activity in TAMR-MCF-7 cells were higher than those in control MCF-7 cells (Figure 3A). Following the exposure of TAMR-MCF-7 cells to an NF-κB inhibitor, 5 or 10 μM tosyl phenylalanyl chloromethyl ketone (TPCK), the protein expression and reporter activity of MAT2A were significantly decreased (Figure 3B and 3C). To confirm the role of NF-κB in MAT2A up-regulation in TAMR-MCF-7 cells, the cells were co-transfected with the MAT2A-luc reporter plasmid and an IκBα overexpression plasmid that suppresses NF-κB activity by blocking the nuclear translocation of NF-κB. As shown in Figure 3D, MAT2A reporter activity was significantly diminished by IkBα overexpression. These findings suggest that persistent activation of NF-κB is critical for the up-regulation of MAT2A in TAMR-MCF-7 cells.

### Down-regulation of miR-146a/b in TAMR-MCF-7 cells and the role of miR-146b in NF-κB-stimulated MAT2A expression

**Figure 4:**
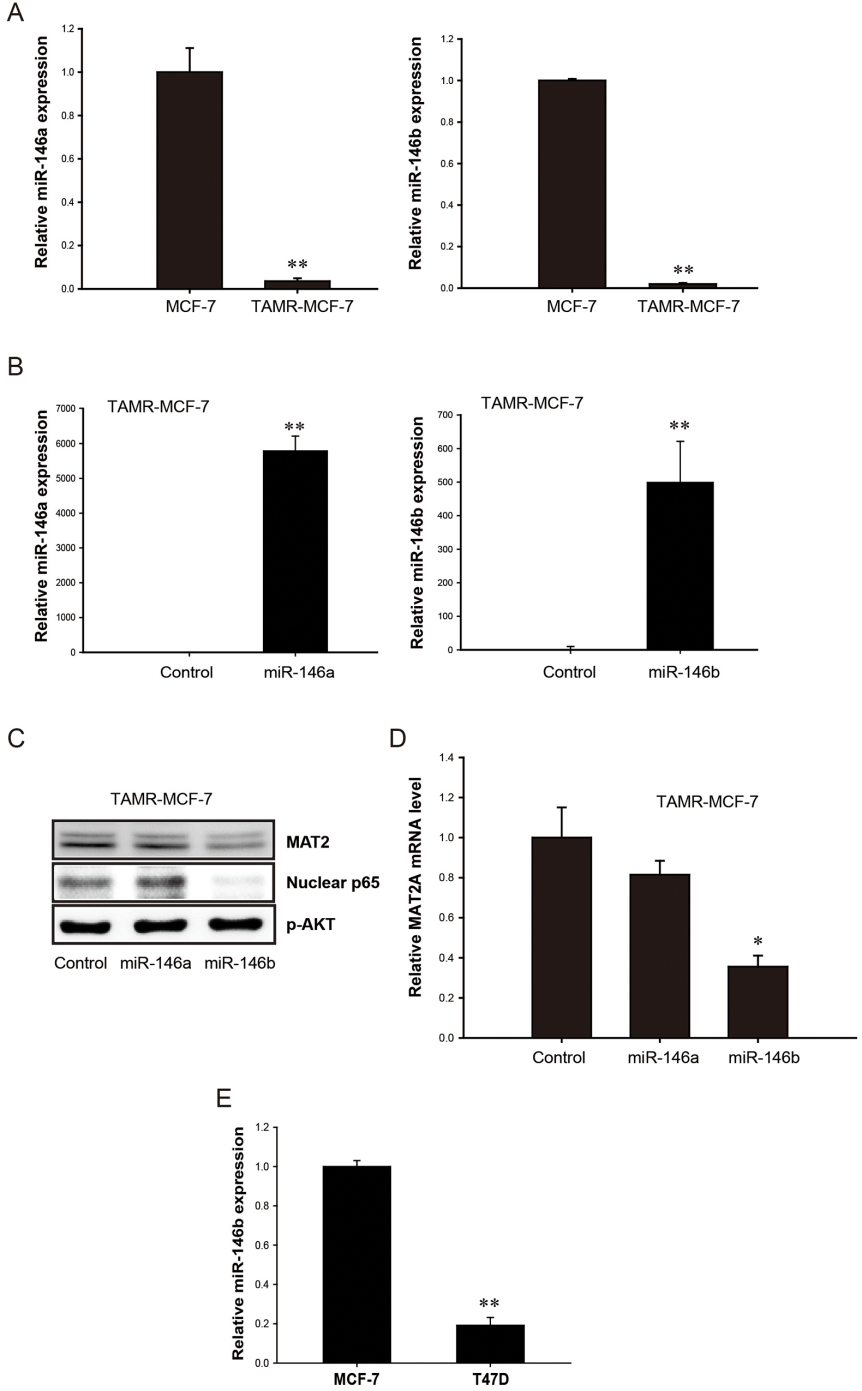
Role of miR-146 family in up-regulation of MAT2A expression in TAMR-MCF-7 cells **A.** Down-regulation of miR-146a and miR-146b expression in TAMR-MCF-7 cells. miR-146a and miR-146b expression in MCF-7 and TAMR-MCF-7 cells were determined by using miScript PCR kit with miScript primers specific for mature miR-146a and miR-146b. Samples were normalized to small nRNA U6. **B.** Effects of miR-146a and miR-146b mimics on the miR-146a/b expression in TAMR-MCF-7 cells. TAMR-MCF-7 cells were cultured in 6 wells plate and then transfected with miR-146a or miR-146b mimics for 36 h (120 *p*mole/well). miR-146a and miR-146b levels were determined by using miScript PCR kit with miScript primers specific for mature miR-146a and miR-146b. Samples were normalized to small nRNA U6. **C.** Effects of miR-146a/b mimic on NF-κB activity and MAT2A protein expression in TAMR-MCF-7 cells. TAMR-MCF-7 cells were cultured in 6 wells plate and then transfected with miR-146a or miR-146b mimics (120 *p*mole/well) for 36 h and immunoblottings were performed. The data were confirmed by two independent experiments. **D.** Effects of miR-146b mimic on MAT2A mRNA expression in TAMR-MCF-7 cells. TAMR-MCF-7 cells were cultured in 6 wells plate and then transfected with miR-146a or miR-146b mimics (120 *p*mole/well) for 36 h. MAT2A mRNA levels were determined by quantitative RT-PCR. Data represent mean ± SD with 3 different samples (significant versus control mimic miR-treated TAMR-MCF-7 cells, *P < 0.05). **E.** miR-146b expression in T47D cells. miR-146b expression in MCF-7 and T47D cells were determined by using miScript PCR kit with miScript primers specific for mature miR-146b. Samples were normalized to small nRNA U6.

As a class of small non-coding RNAs, miRs control the expression of diverse genes through either the inhibition of translational initiation or induction of mRNA degradation [24]. Among various miR families, we focused on miR-146 because miR-146a and -146b function as negative regulators of NF-κB and reduce the metastatic potential of breast cancer cells [25]. Additionally, the overexpression of miR-146a and -146b could suppress NF-κB activity in breast cancer cells. Figure 4A shows the loss of expression of both miR-146a and -146b in TAMR-MCF-7 cells versus control MCF-7 cells. We next hypothesized that a loss of miR-146a or -146b could result in enhanced NF-κB activity and subsequent MAT2A expression in TAMR-MCF-7 cells. When TAMR-MCF-7 cells were transduced with exogenous miR-146a and -146b, the cellular level of miR-146a or -146b was significantly enhanced by 2,896 and 690 fold, respectively, in TAMR-MCF-7 cells (Figure 4B). The overexpression of miR-146b, but not of miR-146a, significantly reduced the nuclear level of p65 in TAMR-MCF-7 cells (Figure 4C). Moreover, the protein and mRNA levels of MAT2A were diminished by miR-146b but not by miR-146a transduction (Figure 4C and 4D). We further compared the level of miR-146b between MCF-7 and T47D cells. As shown in figure 4E, miR-146b expression was reduced in T47D cells (Figure 4E). Our data suggest that miR-146b down-regulation is critical to the enhanced NF-κB activity and MAT2A induction in TAMR-MCF-7 cells.

### Reversal of PTEN promoter methylation by miR-146b in TAMR-MCF-7 cells

**Figure 5:**
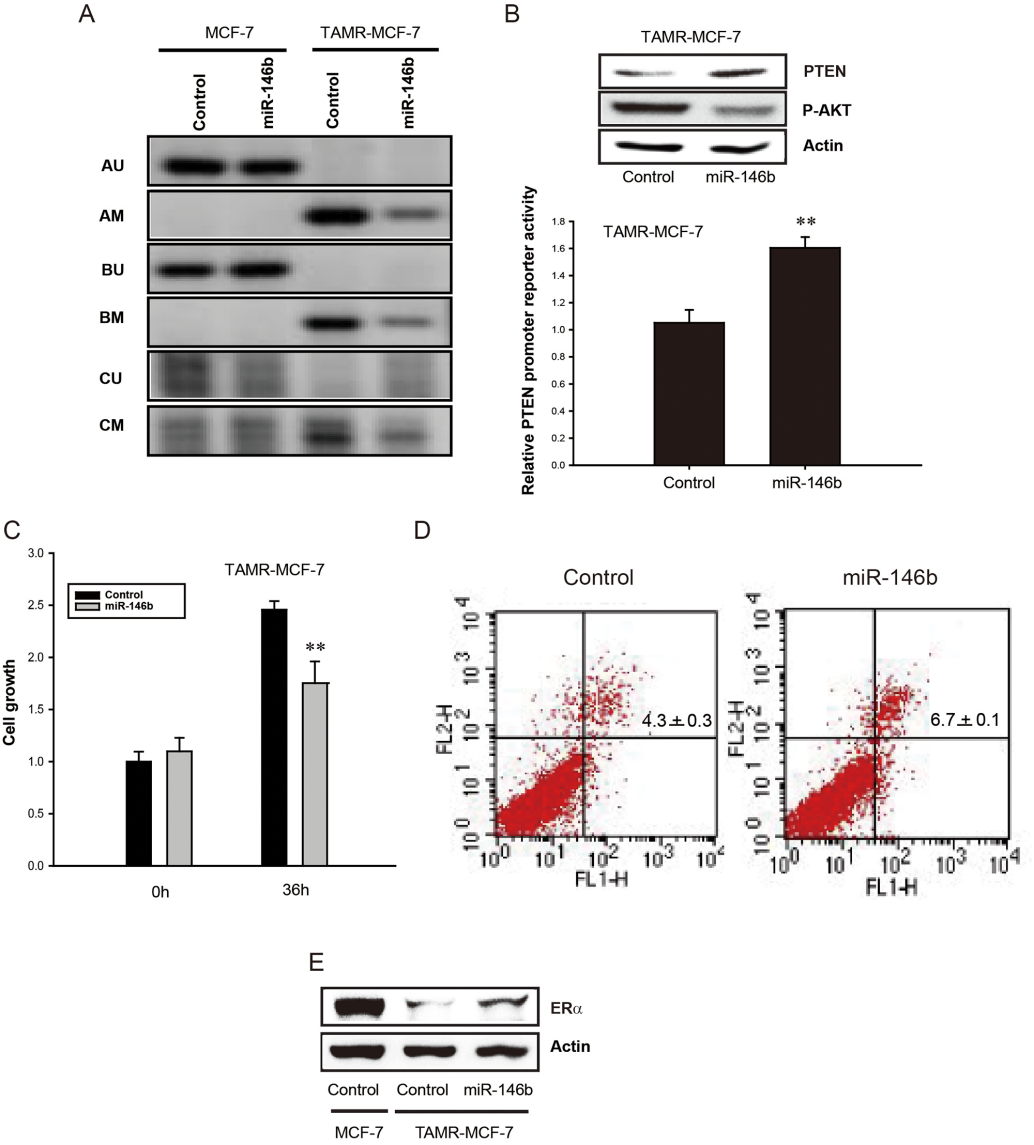
Reversal of PTEN promoter methylation by miR-146b in TAMR-MCF-7 cells **A.** Methylation specific PCR analysis of PTEN gene in MCF-7 and TAMR-MCF-7 cells. Both the cell types were transfected with or without miR-146b mimic (120 *p*mole/well). Methylation specific PCR was performed in three different sites: A, B and C. M and U represent PCR products of methylated and unmethylated allels, respectively. **B.** Upper; Effects of miR-146b mimic on the protein expression of PTEN and phosphorylated Akt. TAMR-MCF-7 cells were transfected with miR-146b mimic for 36 h. PTEN and phosphorylated Akt level were determined from cell lysates by immunoblotting. Lower; Effect of miR-146b mimic on PTEN promoter activity in TAMR-MCF-7 cells. TAMR-MCF-7 cells were co-transfected with PTEN reporter plasmid (0.5 μg/well), phRL-SV plasmids (1 ng/well) and miR-146b mimic (50 *p*mole/well) for 36 h. Luciferase reporter activity was determined described as figure legend of figure 1B. Data represent mean ± SD with 3 different samples (significant versus control mimic miR-treated TAMR-MCF-7 cells, **P < 0.01). **C.** Inhibitory effect of miR-146b mimic on the cell proliferation of TAMR-MCF-7 cells. TAMR-MCF-7 cells were plated in 12 well plates and then transfected with miR-146b mimic (50 *p*mole/well). Cell numbers were counted using automated cell counter (Invitrogen, Carlsbad, CA). Data represent mean ± SD with 3 different samples (significant versus control mimic miR-treated TAMR-MCF-7 cells, **P < 0.01). **D.** Increased tamoxifen sensitivity by miR-146b. TAMR-MCF-7 cells were transfected with miR-146b mimic (120 *p*mole/well) in the presence or absence of 4-hydroxytamoxifen (3 μM) for 36 h. Representative dot plots of TAMR-MCF-7 cells stained with annexin V-FITC (FLH-1) and propidium iodide (FLH-2). Low-right panel represents early apoptotic cells. Upper-left panel and upper-right panel represent late necrotic and apoptotic cells, respectively. Low-left panel represents survival cells. The percentage counts of each quadrant are indicated (*n* = 3). **E.** Effect of miR-146b mimic on ERα expression level in TAMR-MCF-7 cells. TAMR-MCF-7 cells were transfected with miR-146b mimic (120 *p*mole/well) for 36 h, then ERα protein level was determined from total cell lysates by immunoblotting.

We previously revealed that increases in intracellular SAM and DNMT1 expression in TAMR-MCF-7 cells contribute to exaggerated methylation of the PTEN promoter, which causes reduced PTEN expression and enhanced Akt phosphorylation [12]. Because SAM synthesis is exclusively mediated by MAT, we further examined the effect of miR-146b on PTEN promoter methylation. We found that miR-146b transduction in TAMR-MCF-7 cells suppressed aberrant methylation of the PTEN promoter at sites A, B, and C (Figure 5A). Western blot analyses confirmed that miR-146b overexpression increased PTEN expression and, conversely, inhibited Akt phosphorylation in TAMR-MCF-7 cells (Figure 5B, upper). Additionally, PTEN transcription was significantly increased by miR-146b (Figure 5B, lower). Considering our previous findings that PTEN promoter methylation is important for the proliferation and 4-hydroxytamoxifen (active metabolite of TAM) responsiveness of TAMR-MCF-7 cells [12], we further assessed the effects of miR-146b on cell growth and 4-hydroxytamoxifen-induced apoptosis. As expected, miR-146b transduction significantly inhibited cell proliferation (Figure 5C) and potentiated 4-hydroxytamoxifen-mediated apoptosis in TAMR-MCF-7 cells (Figure 5D). These findings confirm that miR-146b decrease is crucial for enhanced cell growth and impaired TAM responsiveness in TAM-resistant breast cancer cells.

One of the most important causes of TAM resistance is dysregulation of ER activity, especially the loss of ERα expression [26]. When we determined ERα expression, ERα level was markedly suppressed in TAMR-MCF-7 cells compared to MCF-7 cells (Figure 5E). Moreover, miR-146b overexpression partially recovered the expression of ERα in TAM-resistant breast cancer cells (Figure 5E).

### Positive feedback between PI3K activation and miR-146b-dependent NF-κB activation in TAMR-MCF-7 cells

**Figure 6:**
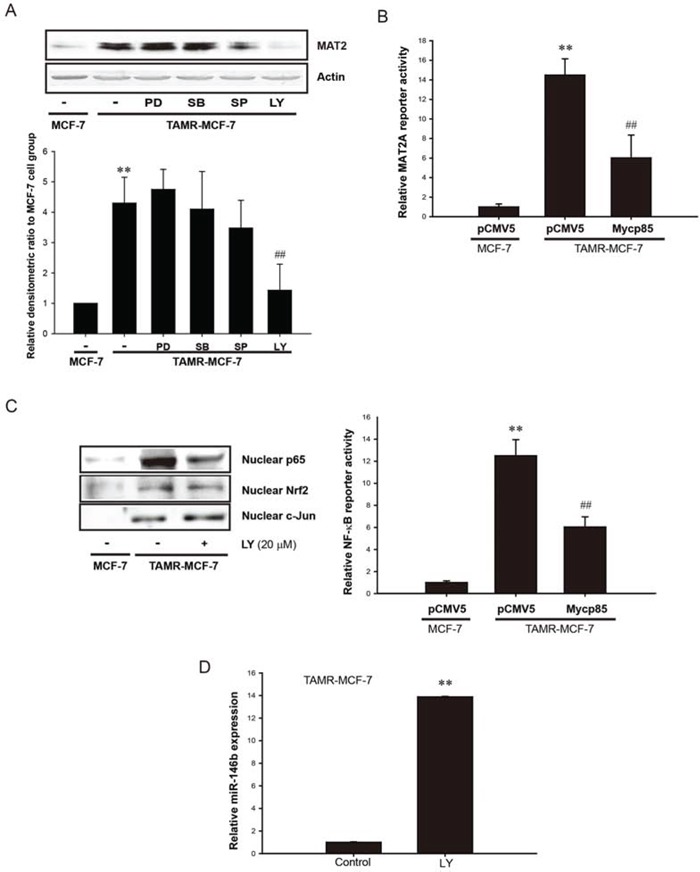
Role of PI3K in NF-κB-dependent MAT2A expression in TAMR-MCF-7 cell **A.** Effect of MAPK inhibitors and PI3K inhibitor on MAT2A expression. Upper; MAT2A protein expression was completely suppressed by 24 h incubation of LY294002 (LY, 20 μM), a PI3K inhibitor in TAMR-MCF-7 cells. PD98059 (PD, 20 μM, ERK inhibitor), SB203580 (SB, 10 μM, p38 kinase inhibitor) and SP600125 (SP, 10 μM, JNK inhibitor) were also used. Lower; Densitometry data. Data represent mean ± SD with 3 different samples (significant versus control MCF-7 cells, **P < 0.01; significant versus TAMR-MCF-7 cells, ^##^*P* < 0.01). **B.** Effect of Mycp85 (dominant negative mutant form of PI3K) overexpression on MAT2A gene transcription. TAMR-MCF-7 cells were co-transfected with MAT2A-luc reporter plasmid (1 μg/well) phRL-SV plasmids (1 ng/well) and pCMV5 or Mycp85 overexpression plasmid (0.5 μg/well) for 18 h in serum free condition. Luciferase reporter activity was determined described as figure legend of Figure 1B. Data represent mean ± SD with 3 different samples (significant versus pCMV5-transfected MCF-7 cells, **P < 0.01; significant versus pCMV5-transfected TAMR-MCF-7 cells, ^##^*P* < 0.01). **C.** Effect of PI3K inhibition on NF-κB activity. Left; nuclear p65, nuclear Nrf2, and nuclear c-Jun detection. TAMR-MCF-7 cells were treated with LY294002 (LY, 20 μM) for 24 h and nuclear levels of p65, Nrf2, and c-Jun were detected by immunoblotting. Right; Effect of Mycp85 overexpression on NF-κB minimal reporter activity. TAMR-MCF-7 cells were co-transfected with pNF-κB-Luc reporter plasmid (1 μg/well) and pCMV5 or Mycp85 overexpression plasmid (0.5 μg/well). Then, pNF-κB-Luc reporter activity was determined 18 h after transfection in serum-free condition. Data represent mean ± SD with 3 different samples (significant versus pCMV5-transfected MCF-7 cells, **P < 0.01; significant versus pCMV5-transfected TAMR-MCF-7 cells, ^##^*P* < 0.01). **D.** Effect of LY294002 (PI3K inhibitor, LY, 20 μM) on miR-146b expression in TAMR-MCF-7 cells. TAMR-MCF-7 cells was exposed to LY294002 for 24 h and then miR-146b level was determined by using miScript PCR. Samples were normalized to small nRNA U6. Data represent mean ± SD with 3 different samples (significant versus vehicle-treated TAMR-MCF-7 cells, **P < 0.01).

Besides miR-mediated post-translational control, MAT2A expression can also be controlled by diverse kinases. To determine the kinase(s) involved in MAT2A transcription, TAMR-MCF-7 cells were incubated for 24 h with mitogen-activated protein kinase family inhibitors, including extracellular signal-regulated kinase (PD98059, 20 μM), p38 kinase (SB203580, 10 μM), c-Jun N-terminal kinase (SP600125, 10 μM), and a PI3K inhibitor (LY294002, 20 μM), and then the MAT2A protein levels were determined. The results showed that only PI3K inhibition selectively decreased MAT2A expression in TAMR-MCF-7 (Figure 6A). To confirm this, we used a dominant-negative mutant of PI3K (Mycp85 overexpression vector). MAT2A-luc reporter activity in TAMR-MCF-7 cells was significantly reduced after co-transfection with Mycp85 (Figure 6B). These results imply that PI3K activation stimulates MAT2A transcription in TAMR-MCF-7 cells. Because NF-κB is a key transcription factor for MAT2A transcription, we next assessed the effects of PI3K inhibition on the enhanced NF-κB activity in TAMR-MCF-7 cells. Both the nuclear translocation of p65 and minimal reporter activity of NF-κB were significantly suppressed by LY294002 treatment and Mycp85 overexpression, respectively (Figure 6C). We also estimated the role of PI3K in c-Jun and Nrf 2 activation in TAMR-MCF-7 cells. PI3K/Akt inhibition did not change the nuclear levels of c-Jun and Nrf 2 in the cell type (Figure 6C). In fact, PI3K signaling is actively involved in the regulation of NF-κB activity in many cell types [27–31].

Next, we addressed the possible crosstalk between miR-146b decrease and PI3K activity. As shown in Figure 5B, miR146b inhibited PI3K activity by blocking methylation of the *PTEN* promoter. When we determined the cellular levels of miR-146a and -146b in TAMR-MCF-7 cells incubated with LY294002 for 24 h, the decreased miR-146b expression was significantly recovered by PI3K inhibition (Figure 6D); however, miR-146a could not be detected after LY294002 treatment (data not shown). These data raise the possibility that a positive feedback loop between miR-146b and PI3K guarantees the sustained activation of NF-κB in TAMR-MCF-7 cells, and that this is essential for the up-regulation of MAT2A and increase in SAM.

## DISCUSSION

Switching from MAT1A to MAT2A expression could contribute to the development of liver cancer [4]. MAT2A expression is associated with rapid cell proliferation and, ultimately, carcinogenesis [4, 6–9]. Although MAT2A function are well known in liver cancer, there has been no reports on its role in breast cancer. Here, we found MAT2A expression was increased in TAMR-MCF-7 cells. Previously, we showed that SAM levels were increased in TAMR-MCF-7 cells compared with MCF-7 cells [12]. Because one of the features of TAMR-MCF-7 cells is a high rate of proliferation, the aberrant increase in MAT2A expression and subsequent increase in SAM synthesis may contribute to the acquisition of a rapid growth phenotype in this cell type.

Until now, limited reports have existed regarding the control of MAT2A expression despite the importance of this gene. Promoter region of human MAT2A contains several potential binding sites for transcription factors, including c-Myb, Nrf2, NF-κB, and AP-1, at −354 to −312 bp in the promoter region, and for Sp1 and activator protein-2 at −73 to −28 bp in the promoter region [13]. A DNase protection assay revealed that protein binding to the specific promoter region responsible for NF-κB, AP-1, c-Myb, or Sp1 was increased in hepatocarcinoma cells compared with normal liver cells [13, 22]. The activation of NF-κB and AP-1 is required for MAT2A up-regulation in TNF-α-treated HepG2 cells, whereas the role of Nrf2, whose binding site partially overlaps with that of AP-1, has not yet been tested [22]. Because we previously demonstrated that the transcriptional activities of NF-κB, AP-1, and Nrf2 were simultaneously enhanced in TAMR-MCF-7 cells [32, 33], we investigated the potential role of these transcription factors. Here, we revealed that MAT2A expression in TAMR-MCF-7 cells is exclusively under the control of NF-κB activity.

miRs are small RNAs that can regulate the expression of genes involved in diverse biological processes, including cell differentiation, cell proliferation, and apoptosis [34, 35]. The loss or mutation of several miRs can contribute to carcinogenesis [36, 37]. Abundant reports have demonstrated that miRs control the function of transcription factors such as NF-κB and Nrf2 in diverse cancer cell types, including leukemia and breast or colorectal cancer [25, 38, 39]. The basal expression level of the miR-146 family is altered in breast cancer cell lines. The miR family consists of miR-146a and -146b, which are distinct genes encoded on chromosomes 5q33 and 10q24, respectively [40]. Recent studies have revealed that miR-146 is a critical factor for cell proliferation and metastasis of diverse cancer types. miR-146a suppresses cell growth, induces cellular apoptosis and inhibits epidermal growth factor receptor downstream signaling in five non-small cell lung cancer cells. FOXP3-induced miR-146a/b prevents tumor cell proliferation and enhances apoptosis in ER-positive breast cancer cells [41]. Especially, miR-146a or − 146b acts as a metastasis suppressor in gastric and triple negative breast cancer cells, which is related with crosstalk between tumor suppressor or oncogenic factor including breast cancer metastasis suppressor 1 and WASF2 [38, 42].

Compared with normal breast epithelial MCF10A cells, the expression of both miR-146a and -146b was decreased in the tumorigenic but weak or non-metastatic cell lines MCF-7, T47D, and MDA-MB-436 [40]. Moreover, a further decrease was found in metastatic MDA-MB-435 cells [40]. Particularly, because miR-146 is known to be a regulator of NF-κB activity [25, 43], we focused on the role of the miR-146 family in NF-κB-stimulated MAT2A expression in TAMR-MCF-7 cells. The expression levels of both miR-146a and -146b were diminished in TAMR-MCF-7 cells compared with MCF-7 cells. We further found that both MAT2A expression and the nuclear translocation of NF-κB/p65 in TAMR-MCF-7 cells were suppressed by miR-146b overexpression but not by miR-146a. These findings support the role of NF-κB in MAT2A gene transcription and suggest the underlying mechanism of enhanced NF-κB activity to be related to miR-146b down-regulation in TAM-resistant breast cancer. TRAF6 and IRAK1, two key adaptors in Toll-like receptor or cytokiner/NF-κB signaling, are known as direct targets of miR-146a and miR-146b. Despite their functional similarities, miR-146a and miR-146b are distinct genes encoded on different chromosomes and controlled by different mechanisms depending on cell type and the environment [40]. It would be possible that miR-146a-mediated inactivation of TRAF6/IRAK1-NF-κB signaling is impaired in TAMR-MCF-7 cells.

**Figure 7:**
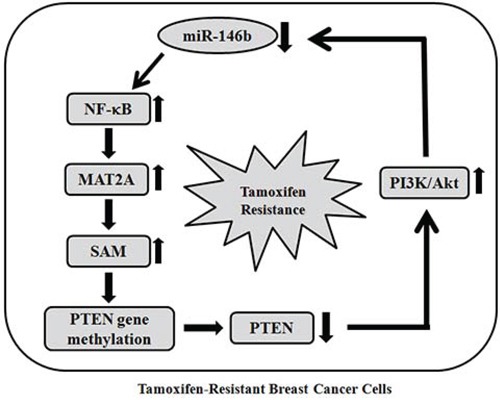
Proposed mechanism for TAM-resistance The scheme shows positive feedback loop between the PTEN-controlled PI3K/Akt pathway and miR-146b-controlled NF-κB/MAT2A expression.

Several tumor-suppressive genes such as BRCA1, TIMP1 and ERα are hypermethylated in breast cancer [44]. When we further determined methylation status of ERα and BRCA1 gene, methylation of CpG ER1 region of ERα gene and BRCA1 promoter were increased in TAMR-MCF-7 cells compared to MCF-7 cells (Supplemental 2). We previously showed that the loss of PTEN expression in TAMR-MCF-7 cells was due to hypermethylation of the *PTEN* promoter caused by an abnormal increase in SAM synthesis [12]. Because MAT2 functions exclusively in SAM synthesis, we further checked the methylation status of the *PTEN* promoter in miR-146b-overexpressing TAMR-MCF-7 cells. The overexpression of miR-146b reduced the methylation intensity of the *PTEN* promoter and restored the expression of PTEN in TAMR-MCF-7 cells. This suggests that miR-146b-dominated MAT2A expression is crucial for SAM synthesis and subsequent PTEN expression in TAMR-MCF-7 cells. PTEN is a negative regulator of the PI3K/Akt pathway, which determine the cell growth, survival, and inhibition of apoptosis in both normal and cancer cells [45–47]. Additionally, the sustained activation of PI3K/Akt is considered to be a key event in the development of TAM resistance in breast cancer cells [48–50]. In TAMR-MCF-7 cells, basal PI3K/Akt activity was completely reversed by miR-146b, and we elucidated the capability of miR-146b to inhibit cell growth and increase apoptotic sensitivity to 4-hydroxytamoxifen in TAMR-MCF-7 cells. Our data imply that miR-146b decrease is essential for NF-κB-stimulated MAT2A induction and the control of cell proliferation and apoptosis in TAMR-MCF-7 cells. Interestingly, PI3K inhibition by LY294002 treatment could also suppress both MAT2A expression and NF-κB activation in TAMR-MCF-7 cells. Moreover, the decrease in miR-146b expression was significantly recovered by PI3K inhibition. These results imply that constitutive activation of the PI3K/Akt pathway is also involved in the control of miR-146b-mediated MAT2A expression in TAMR-MCF-7 cells. Hence, a positive feedback loop between the PTEN-controlled PI3K/Akt pathway and miR-146b-controlled NF-κB/MAT2A expression in TAMR-MCF-7 cells seems to be crucial for the enhanced cell proliferation and decreased TAM responsiveness (Figure 7). Although the case number is limited, we showed that MAT2A immunoreactivity was significantly higher in TAM-resistant cases of human breast cancer than in TAM-sensitive cases. Therefore, miR-146b decrease and MAT2A induction could be new phenotypes contributing to the growth of breast cancer cells and tamoxifen resistance in breast cancer cells.

## MATERIALS AND METHODS

### Materials

Antibodies against MAT1, MAT2A, Nrf2, p65, c-Jun, c-Fos, Jun B, Jun D, PTEN were purchased from Santa Cruz Biotechnology (Santa Cruz, CA). Antibodies specific for phosphorylated Akt and Akt were obtained from Cell Signaling Technology (Beverly, MA, USA). Horseradish peroxidase-conjugated donkey anti-rabbit, anti-goat IgG, and alkaline phosphatase-conjugated donkey anti-mouse IgG were acquired from Jackson Immunoresearch Laboratories (West Grove, PA). Anti-actin antibody and most of the reagents used for molecular studies were obtained from Sigma (St. Louis, MO). The MAT2A-luc reporter plasmid was kindly donated by Dr. SC Lu (University of Southern California, CA). An overexpression plasmid for inhibitor of κBα (IκBα) and a pGL-ARE minimal reporter (containing three copies of the ARE region of the quinone oxidoreductase promoter) were donated by Dr. KY Lee (Chonnam National University, Korea) and Dr. MK Kwak (Catholic University, Korea), respectively. pNF-κB-luc and pAP-1-luc reporter plasmids were purchased from Stratagene (La Jolla, CA). Mimic hsa-miR-146b-3p (Dharmacon, C30109201), has-miR-146a (Dharmacon, C30063003) or mimic negative control (Dharmacon, CN0010000105) were supplied from Thermo Fisher Scientific (Lafayette, CO).

### Cell culture and establishment of TAMR-MCF-7 cells

MCF-7 and MDA-MB-231 cells were cultured at 37°C in 5% CO_2_/95% air in Dulbecco's modified Eagle's medium (DMEM) containing 10% fetal bovine serum (FBS), 100 units/ml penicillin, and 100 μg/ml streptomycin. TAMR-MCF-7 cells were established using methods previously reported [12, 14, 15]. T47D cells were cultured in RPMI-1640 medium containing 10% FBS, 100 units/ml penicillin, and 100 μg/ml streptomycin.

### Preparation of the nuclear fraction and immunoblot analysis

Nuclear extracts preparation and sodium dodecylsulfate-polyacrylamide gel electrophoresis and immunoblot analysis were performed according to previously published procedures [16].

### Reporter gene analysis

A dual-luciferase reporter gene assay (Promega, Madison, WI) was used to determine promoter activity. Briefly, cells were plated in 12-well plates and transiently transfected with 1 μg/ml reporter plasmids and phRL-SV plasmid (hRenilla luciferase expression for normalization) using Hillymax^®^ reagent (Dojindo Molecular Technologies, Gaithersburg, MD). The cells were then incubated in culture medium without serum. Firefly and hRenilla luciferase activities in the cell lysates were measured using a luminometer (LB941, Berthold Technologies, Bad Wild, Germany). Relative luciferase activities were calculated by normalizing the promoter-driven firefly luciferase activity to the hRenilla luciferase.

### Immunohistochemistry for human cancer tissues

Blocks for all the samples were consecutively cut in 4 μm sections and mounted on poly-l-lysine coated glass slides. Xylene was used to remove the paraffin from the sections, and the samples were rehydrated. Antigen retrieval was performed by boiling sections for 5 min in 1 μM sodium citrate buffer (pH 6.0) in a microwave oven. Endogenous peroxidase activity was blocked using 3% hydrogen peroxide in methanol for 10 min, followed by three times washing with PBS. Sections were then incubated overnight with anti-MAT2A antibody (NBP1-28605; Novus biological, CO, USA) at 4°C. After washing with PBS, sections were incubated with HRP-conjugated anti-rabbit IgG for 30 min and washed with PBS. The color was developed by incubation with DAB solution. Finally, sections were counterstained with hematoxylin, dehydrated, mounted, and observed.

### miR quantitative polymerase chain reaction (PCR)

miR expression was determined by collecting from total RNA from 70% to 90% confluent cells. Total RNA was isolated by using Trizol (Invitrogen, USA) and was then converted to cDNA by using the miScript reverse transcription kit (Qiagen, Valencia, CA). miScript primers specific for mature miRNAs were hsa-miR-146a (5′-CCTCTGAAATTCAGTTCTTCAG-3′) and hsa-miR-146b (5′-TGCCCTGTGGACTCAGTTCTGG-3′)(Bioneer, Eumsung, Korea). Real time PCR was performed using miScript PCR kit. All samples were normalized to small nRNA U6 to calculate the fold changes.

### Quantitative PCR

Total RNA was isolated by using Trizol (Invitrogen, USA). qPCR was performed with MiniOpticon real time PCR detection system (Bio-Rad laboratories Inc., Hercules, CA) and primers specific for MAT2A (Forward, 5′-GTGGCAGATTTGTTATTGGTG-3′; Reverse, 5′-CAACGAGCAGCATAAGCA-3′) and S16r (Forward, 5′-TCCAAGGGTCCGCTGCAGTC-3′; Reverse, CGTTCACCTTGATGAGCCCA-3′).

### Methylation-specific PCR

The methylation status of the PTEN, BRCA1 and ERα promoter was determined by methylation-specific PCR after bisulfite-modification. The methylation sites of PTEN gene promoter included three regions (A, B, and C regions) [12, 17]. CpG ER1 region of ERα gene and BRCA1 promoter were also determined [18, 19]. Genomic DNA was isolated and modified by bisulfite using an EpiTect Bisulfite kit (Qiagen, Valencia, CA) and methylated and unmethylated genomic regions can be amplified by PCR using each sequence-specific pairs of primers [17].

### Cell proliferation

Cells were seeded into 12 well plates and 50% confluent cells were transiently transfected with 50 *p*mole/well mimic hsa-miR-146b-3p or mimic negative control and the transfected cells was then incubated in DMEM containing 5% charcoal-stripped FBS for additional 36 h. Media were then removed and the transfected cells were harvested with trypsin, stained with 0.4% trypan blue. Cell numbers were counted using automated cell counter (Invitrogen, Carlsbad, CA).

### Flow cytometry

TAMR-MCF-7 cells were plated in 6 well plates and transfected with 120 *p*mole/well mimic hsa-miR-146b-3p or mimic negative control. The transfected cells were incubated in the presence or absence of 3 μM 4-hydroxytamoxifen in serum free medium for 36 h. The cells were harvested with trypsin and stained with both annexin V-fluorescein isothiocyanate (FITC) and propidium iodide according to ApopNexin FITC apoptosis detection Kit (Millipore, Temecula, CA), and analyzed by flow cytometry (FACStar, BDBiosciences, Mississauga, ON) set for FLH-1 (annexin V) and FLH-2 (propidium iodide). Total 10^4^ cells were counted for each sample.

### Statistical analysis

Scanning densitometry was performed using an LAS-3000 mini (Fujifilm, Tokyo, Japan). Student's *t*-test or one-way analysis of variance (ANOVA) followed by Newman–Keuls multiple comparison test were used to examine between group differences. Statistical significance was accepted at either *P* < 0.05 or *P* < 0.01.

## SUPPLEMENTARY FIGURES



## Abbreviations

AP-1: Activator protein-1
DNMT: DNA methyltranferase
ERα: Estrogen receptor-α
IκBα: Nuclear factor of κB inhibitor α
MAT: Methionine adenosyltransferase
miR: microRNA
NF-κB: Nuclear factor-κB
Nrf2: NF-E2-related factor2
PI3K: Phosphoinositide 3-kinase
PTEN: Phosphatase and tensin homolog
SAM: S-adenosylmethionine
siRNA: Small interfering RNA
TPCK: Tosyl phenylalanyl chloromethyl ketone
