# Supplementary material for: Induction of methionine adenosyltransferase 2A in tamoxifen-resistant breast cancer cells
Source: Oncotarget. 2015 Sep 18;7(12):13902–16. doi: 10.18632/oncotarget.5298 (PMC4924687; doi:10.18632/oncotarget.5298)
Supplement: Supplementary file 1 [file oncotarget-07-13902-s001.pdf]

SUPPLEMENTARY FIGURES

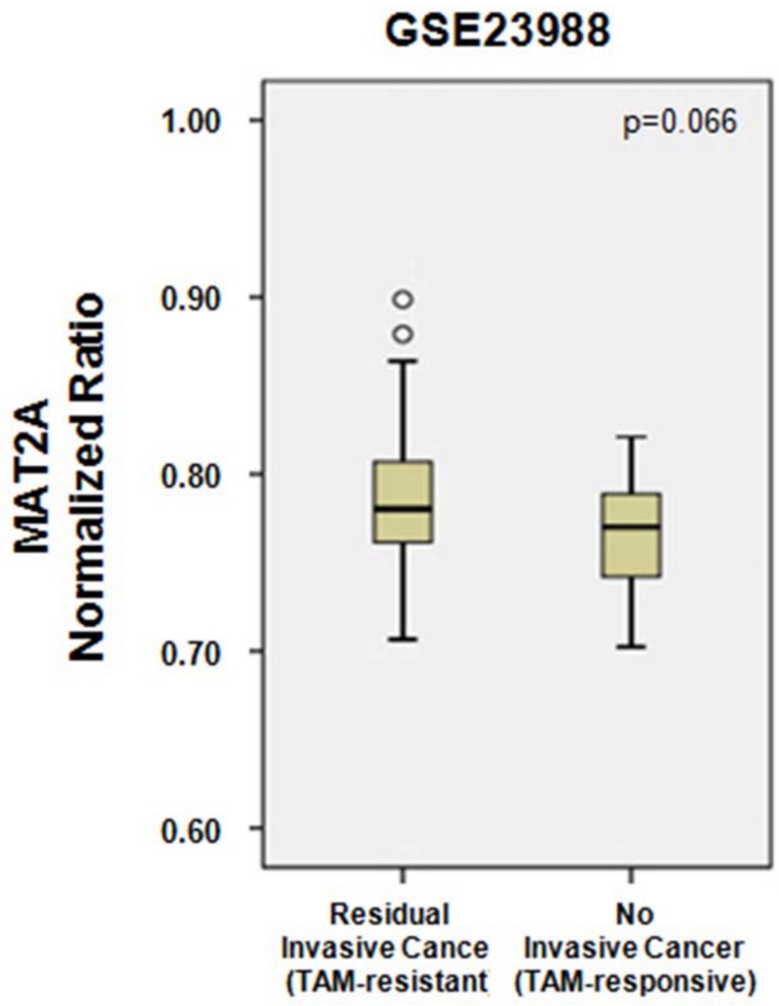

Supplementary Figure S1: GEO MAT2A gene expression analysis. The gene expression date was downloaded from Gene Expression Omnibus (GEO, http://www.ncbi.nlm.nih.gov/geo). The accession number was GSE23988, providing 62 pre-chemotherapy biopsies of HER2 normal breast cancer patients (ER-positive and ER-negative subtypes) with the results of the TAM- chemotherapy. Series matrix file was matched the probes on platform GLP96, excluded 5 normal samples, rearranged into groups according to existence of residual invasive cancer and normalized by a control gene. The Differentially Expressed Gene was identified by two tailed t-test.

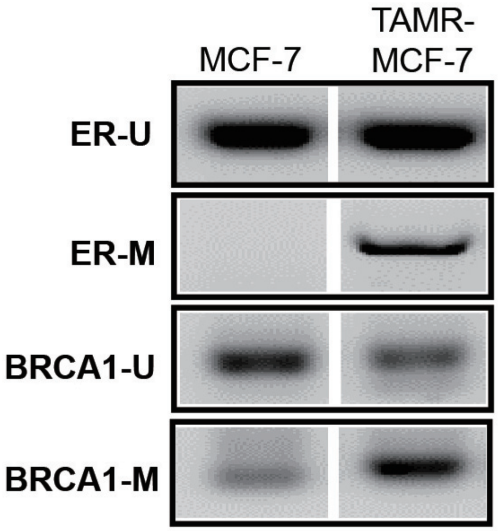

Supplementary Figure S2: Methylation specific PCR analyses of CpG ER1 region of ERα gene and BRCA1 promoter in MCF-7 and TAMR-MCF-7 cells. M and U represent PCR products of methylated and unmethylated alleles, respectively.
